# Underdiagnosis and diagnostic delay in chronic inflammatory demyelinating polyneuropathy

**DOI:** 10.1007/s00415-020-10287-7

**Published:** 2020-11-10

**Authors:** Umair J. Chaudhary, Yusuf A. Rajabally

**Affiliations:** 1 Inflammatory Neuropathy Clinic, Department of Neurology, Queen Elizabeth Hospital, University Hospitals Birmingham NHS Foundation Trust, Birmingham, B15 2TH UK; 2 Aston Medical School, Aston University, Birmingham, UK

**Keywords:** Chronic inflammatory demyelinating polyneuropathy, Diagnostic delay, Guillain–barre syndrome, Underdiagnosis

## Abstract

**Background:**

The frequency and causes of underdiagnosis of chronic inflammatory demyelinating polyneuropathy (CIDP) are uncertain. We aimed to assess the frequency and electroclinical features of pre-referral CIDP underdiagnosis and the duration of delay prior to diagnosis and treatment initiation in a tertiary specialist clinic.

**Methods:**

We retrospectively investigated 60 consecutive patients attending our Inflammatory Neuropathy Service, between 2015 and 2019, with a final diagnosis of treatment-responsive definite/probable CIDP. We reviewed the clinical and electrophysiological data in light of European Federation of Neurological Societies/Peripheral Nerve Society (EFNS/PNS) guidelines and determined the frequency, causes and delay in diagnosis of CIDP.

**Results:**

An initial alternative diagnosis to that of CIDP had been made in 68.3% (41/60) of patients. The commonest alternative diagnosis was of Guillain–Barré syndrome (GBS) in 23.3% (14/60) patients. Non-GBS underdiagnoses (27/60; 45%) mainly consisted of genetic neuropathy (8/27; 29.6%), diabetic neuropathy (5/27; 18.5%) and chronic idiopathic axonal polyneuropathy (4/27; 14.8%). Non-GBS underdiagnoses were predominantly due to non-recognition of proximal weakness (70.4%), multifocal deficits (18.5%) or proprioceptive loss (7.4%). Electrophysiological misinterpretation was contributory to pre-referral non-GBS underdiagnoses of CIDP in 85% of patients. Mean diagnostic delay in patients with non-GBS underdiagnoses of CIDP was of 21.3 months (range 2–132 months).

**Conclusion:**

Underdiagnosis of CIDP is frequent and may lead to significant diagnostic and treatment delay. We suggest that lack of comprehensive and precise attention to typical electroclinical features of CIDP and its diagnostic criteria at the time of initial evaluation are equally contributory to underdiagnoses.

## Introduction

Chronic inflammatory demyelinating polyneuropathy (CIDP) is a progressive motor and/or sensory neurological condition affecting peripheral nerves. The prevalence of CIDP ranges between 1.6 and 7/100,000 population and may vary based on the diagnostic criteria used [1, 2]. CIDP can have multiple relapses causing significant disability in affected population if left untreated. Hence, timely and correct diagnosis and treatment is of paramount importance.

The issue of overdiagnosis of CIDP i.e., patients receiving an erroneous diagnosis of CIDP and treated as such, has been the focus of attention in recent years. Potential causes of overdiagnosis with a wide range of alternative diagnoses have been studied by a North-American group [3]. The delay in diagnosis of CIDP has been reported to range from 2 to 64 months [4]. A more recent Dutch study showed diagnostic delay of more than 12 months in 26% of cases [5]. However, the precise frequency and the factors leading to underdiagnoses (i.e., patients receiving an initial erroneous non-CIDP diagnosis and treated as such and having a final diagnosis of CIDP) of treatment-responsive CIDP is unknown, which in turn may have major consequences for offering immunomodulatory therapy to potentially eligible patients at an early stage when it is most likely to be effective.

We aim to ascertain, the frequency and electroclinical features leading to underdiagnoses of CIDP prior to attending a tertiary specialist neuropathy clinic i.e., pre-referral underdiagnoses, and the duration of resulting diagnostic and treatment delay in patients with treatment-responsive CIDP.

## Methods

We retrospectively reviewed our electronic institutional records of all patients with a diagnosis of CIDP attending our Specialist Inflammatory Neuropathy Service, Queen Elizabeth Hospital, Birmingham, UK. Sixty patients fulfilling the electroclinical diagnostic criteria of CIDP as per European Federation of Neurological Societies/Peripheral Nerve Society (EFNS/PNS) Guidelines, for “definite” or “probable” CIDP [6], and having demonstrated objective clinical improvement after immunotherapy between March 2015 and June 2019 were selected. This study was reviewed and approved by our relevant institutional review board (CARMS no. 15354, July 2019).

Treatment response was defined by a 1-point improvement of the Overall Neuropathy Limitation Score (ONLS) [7, 8], except for a change from 1 to 0 on the upper limb scale, as not functionally relevant. In addition, and for the purposes of the current analysis, improvement of the inflammatory Rasch-built Overall Disability Scale [9] by 4 raw points (out of 48) or of Jamar grip dynamometry by > 5 kg [10], or of an MRC sum score improvement by at least 4 points [8], as well as improvement of the timed 10-m walk by at least 25% [11], were considered as indicative of treatment response, in absence of change of the ONLS. We acknowledge the limitation that some of these scales are not validated specifically in clinical practice, but believe they are the best currently available.

Detailed clinical records for each patient were reviewed for demographics, presenting complaints, examination findings, initial pre-referral diagnoses, and electrophysiological reports. Cerebrospinal fluid (CSF) examination and nerve imaging were performed at different times in the disease course and at different centres and were excluded from the current analysis.

We calculated the frequency of pre-referral diagnosis and alternative diagnoses other than that of CIDP. To calculate the frequency of pre-referral underdiagnoses, delay to final diagnosis of CIDP and delay to receive first treatment, we excluded cases of acute or subacute onset, initially diagnosed with Guillain–Barré syndrome (GBS) or subacute inflammatory demyelinating polyradiculoneuropathy (SAIDP), as we considered these were due to unexpected clinical progression rather than diagnostic error.

For each patient, we performed a detailed review of clinical records to ascertain the predominant clinical feature supporting the diagnosis of CIDP. We focussed on four common clinical features of CIDP based on EFNS/PNS criteria [6] which could have potentially contributed to pre-referral underdiagnoses: (i) presence of proximal weakness (for typical CIDP) (ii) presence of multifocal motor and sensory deficits (for Lewis Sumner syndrome (LSS)), (iii) presence of proprioceptive involvement (for typical and sensory forms) and (iv) presence of chronic severe symmetric tetraparesis majorly impacting on upper and lower limb function (for severe forms of typical CIDP). We did not include tendinous areflexia in the abovementioned clinical features as all patients had absent deep tendon reflexes.

For each patient, we reviewed pre-referral electrophysiological studies for the actual data and waveforms, as well as the available interpretation and conclusion. We requested repeat of the electrophysiology studies at our institution (excluding those patients who already had electrophysiology studies within our unit). We then, independent of all reports, established fulfilment of EFNS/PNS criteria. Hence, we concurrently also identified the omissions and inaccuracies in the clinical neurophysiologist’s reports which had contributed to underdiagnoses. We focussed on 4 points derived from EFNS/PNS criteria [6] as previously applied in validation studies [12, 13]: (i) consideration of motor conduction slowing and distal motor latency prolongation for two nerves (ii) consideration of conduction block, temporal dispersion, F-wave analysis, for at least two nerves (iii) mention of using a set of electrodiagnostic criteria, or cut-offs to define significant abnormality (iv) mention of “acquired demyelinating neuropathy” or “CIDP. Presence of any 3 of these 4 points in the report was considered to be diagnostic of CIDP electrophysiologically.

## Results

**Fig. 1:**
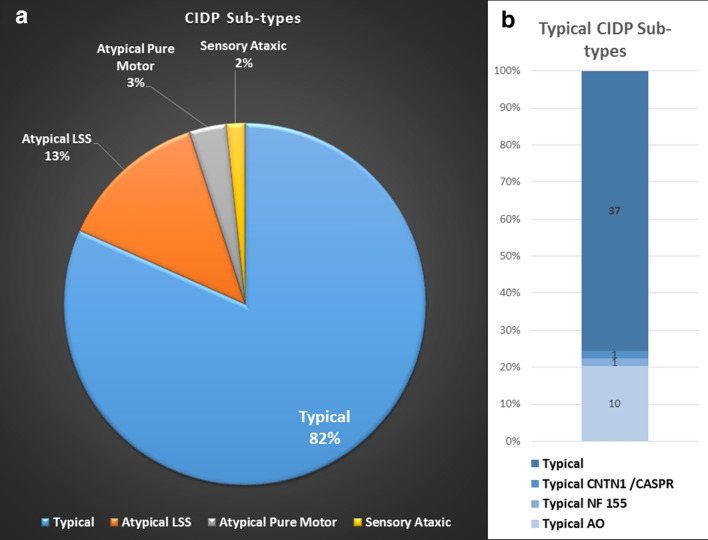
CIDP subtypes. *CIDP* chronic inflammatory demyelinating polyneuropathy, *LSS* Lewis Sumner syndrome, *AO* acute onset

There were 19 females and 41 males. Mean age was 61.7 years (range 24–86). Fifty patients were referred by neurologists, one by a neurophysiologist, three by general physicians and six by other specialists, after initial assessment. Fifty six (93.3%) had “definite” CIDP and four (6.7%) had probable” CIDP. Forty nine out of sixty (82%) patients had typical CIDP sub-type, 8/60 (13.3%) had atypical LSS variant, 2/60 (3%) had atypical pure motor variant, and one had sensory ataxic variant (see Fig. 1). Out of 49 patients with typical CIDP, 10 (20%) had acute onset variant, and one each had feature with NF155 or CNTN1 / CASPR antibodies positive. Effective treatment administered for CIDP was intravenous immunoglobulin in 37 patients (61.7%), intravenous steroids in 16 (26.7%), plasma exchange in 5 (8.3%) and first-line treatment combinations in 10 (16.7%). Four patients (6.7%) had been effectively treated with rituximab. All patients had objective improvement in post immune-modulatory treatment, as per criteria defined in methods section.

**Fig. 2:**
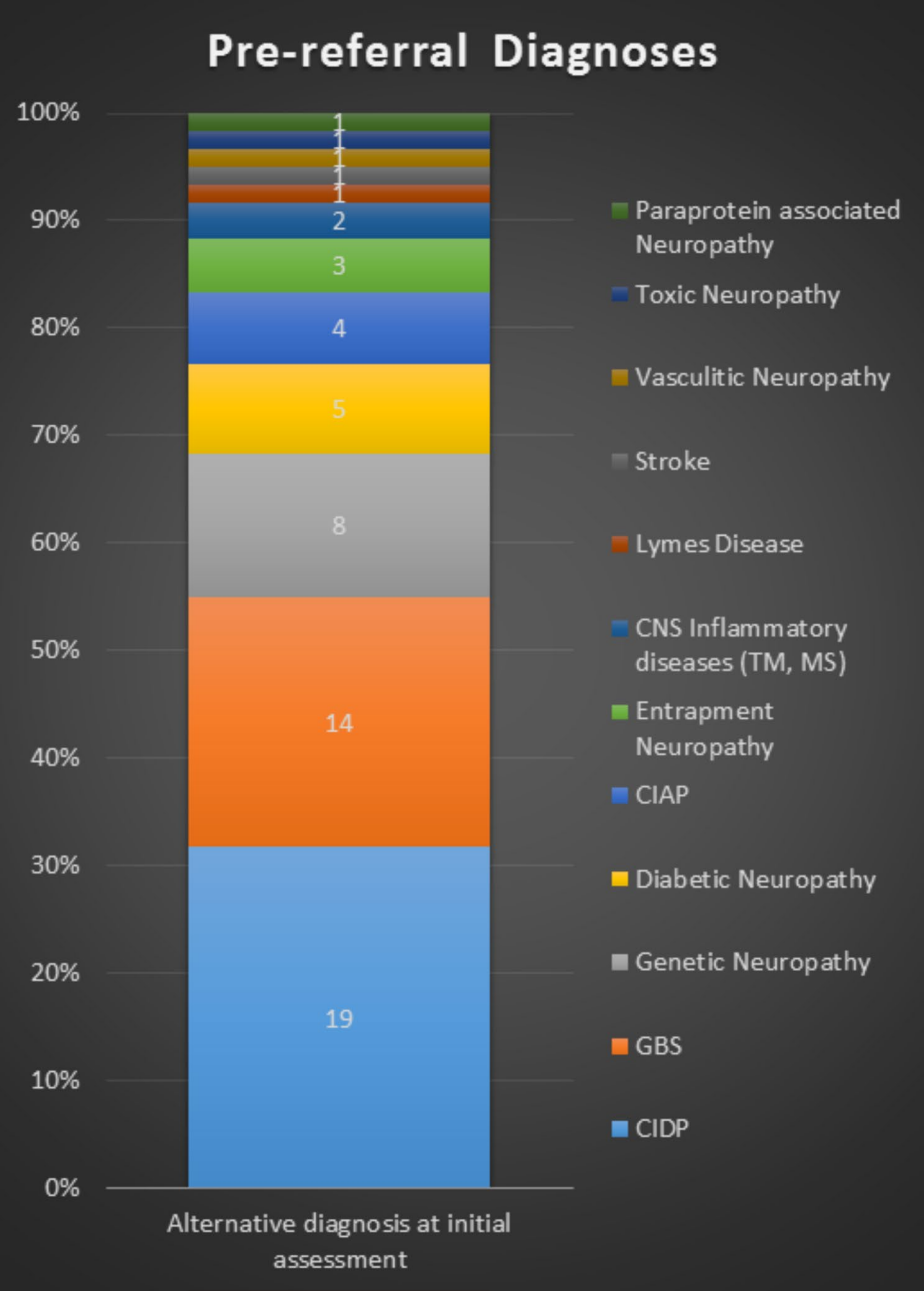
Pre-referral diagnosis for CIDP patients. *CIDP* chronic inflammatory demyelinating polyneuropathy, *GBS* Guillain–Barré syndrome, *CIAP* chronic idiopathic axonal polyneuropathy, *TM* transverse myelitis, *MS* multiple sclerosis

Nineteen patients (19/60; 31.6%) received a pre-referral diagnosis of CIDP. Forty one of the 60 patients (68.3%) had received an alternative pre-referral diagnosis other than that of CIDP (see Fig. 2). The most frequent pre-referral diagnosis was GBS in 14/60 (23.3%) patients. Most of these cases were treated initially at our centre and the diagnosis rectified to one of acute onset CIDP, during or shortly after hospital stay, after three or more relapses, extending beyond 8 weeks from onset. In one case, CIDP was diagnosed with a 12-month delay, after a partially treatment-responsive acute-onset disease treated outside our centre, with subsequent progression.

Twenty seven out of 60 (45%) patients, at the time of pre-referral assessment, had non-GBS underdiagnoses. The most frequent non-GBS underdiagnoses was that of genetic neuropathy in 8/27 patients (29.6%). All patients with a pre-referral diagnosis of genetic neuropathy had genetic testing for CMT, HNPP and, if negative, our institutional CMT gene panel which includes MPZ and GJB1, as part of diagnostic work up. Two patients had genetically confirmed CMT1A, one had a genetically confirmed MPZ mutation, and the remaining five had negative genetics. However, the three patients with genetically confirmed CMT were diagnosed with “definite” co-existent CIDP in view of rapid recent deterioration, proximal weakness and proprioceptive sensory loss, electrophysiological features of acquired inflammatory neuropathy (conduction block and excessive temporal dispersion), and objective response to immunotherapy. The remaining five patients, for their part, all had CIDP clinically and electrophysiologically.

Five out of 27 (18.5%) patients had a pre-referral diagnosis of non-CIDP neuropathies of diabetes. This included diabetic polyneuropathy in 4 (14.8%) and diabetic lumbosacral radiculo-plexus neuropathy in 1 (3.7%). In 4/27 (14.8%), the pre-referral diagnosis was one of chronic idiopathic axonal polyneuropathy (CIAP). Vasculitic neuropathy was the initial diagnosis in 1/27 (3.7%), compressive focal entrapment in 3/27 (11.1%), toxic neuropathy in 1/27 (3.7%), paraprotein associated neuropathy, considered unrelated to CIDP, in 1/27 (3.7%), and Lyme’s disease with polyradiculopathy in 1/27 (3.7%). Non-neuropathic conditions were initially diagnosed in three patients (11.1%): stroke in one and CNS inflammatory disease in two.

We found that 16/19 (84.2%) patients with pre-referral CIDP diagnoses had typical clinical features of CIDP as per EFNS/PNS criteria. Two patients (10.5%) had atypical pure motor weakness, and one (5.2%) patient had atypical LSS with multifocal sensori-motor symptoms. In comparison, 19/27 (70.3%) patients with a pre-referral non-GBS underdiagnoses had typical clinical features of CIDP including proximal weakness or loss of proprioception. Seven patients (25.9%) had atypical LSS with multifocal sensori-motor symptoms and one (3.7%) had atypical sensory ataxic CIDP. Hence, although LSS was more frequent in the underdiagnosed than the correctly diagnosed group, this did not reach statistical significance, possibly due to small numbers included (7/27 vs. 1/19; p = 0.12).

**Fig. 3:**
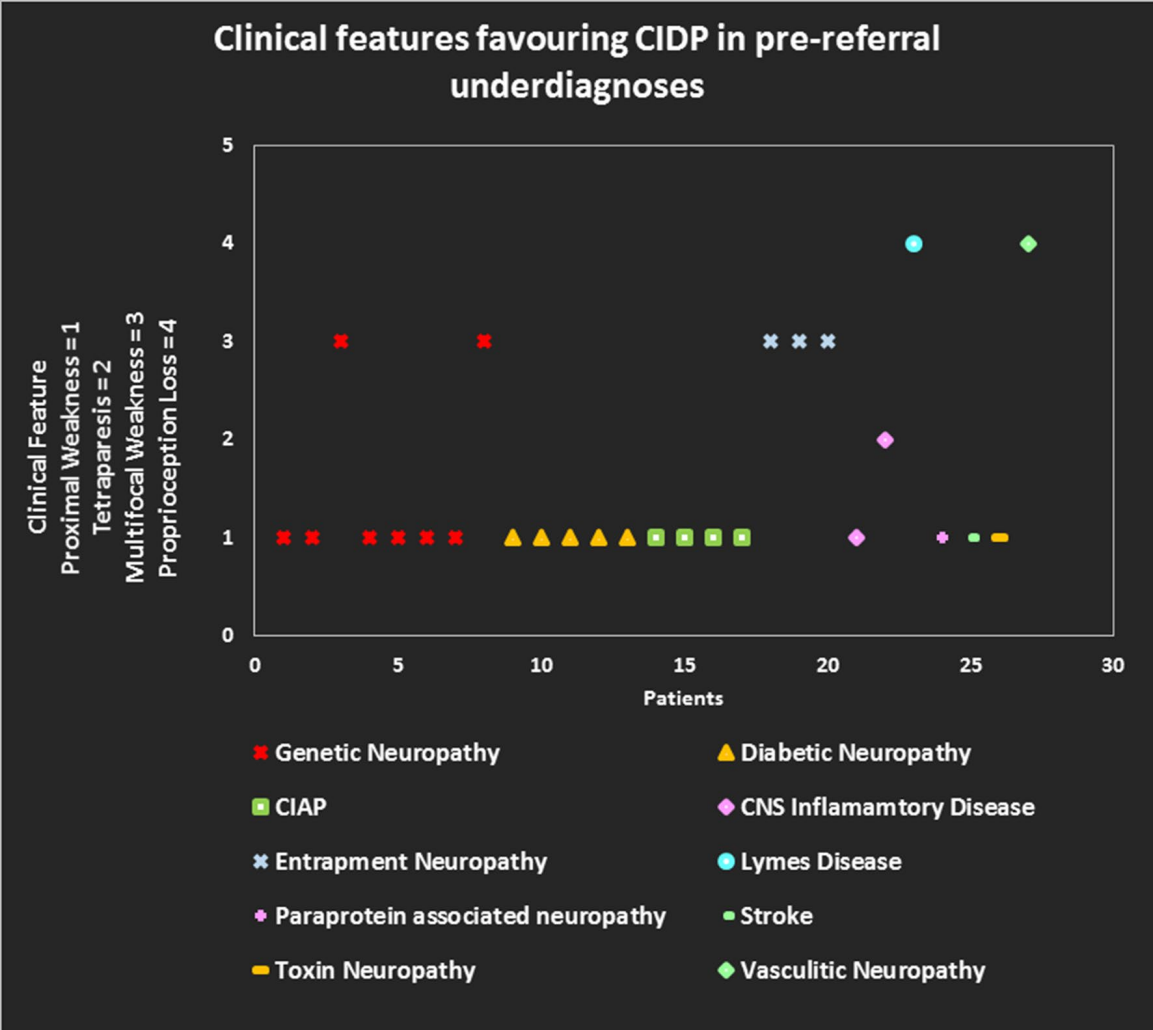
Clinical feature favouring CIDP in pre-referral underdiagnoses. *CIDP* chronic inflammatory demyelinating polyneuropathy, *CIAP* chronic idiopathic axonal polyneuropathy

In 27 patients with pre-referral non-GBS underdiagnoses, the main clinical features in favour of CIDP, which might have been discounted at the time of pre-referral assessment, were: proximal weakness in 19/27 (70.4%), multifocal motor and sensory deficit in 5/27 (18.5%), proprioceptive sensory loss in 2/27 (7.4%) and tetraparesis in 1/27 (3.7%) (Fig. 3).

In 23/27 (85.2%) patients with pre-referral non-GBS underdiagnoses, electrophysiology reports were contributory to underdiagnosis, as 3 of the 4 electrophysiology points, derived from EFNS/PNS criteria as detailed in “Methods”, were not met. In detail (see Table 1), 19/27 (70.4%) patients met only one point, 4/27 (14.8%) met two points, 1/27 (3.7%) met 3 points and 3/27 (11.1%) met all 4 points. Thus, in only four patients (14.8%), did pre-referral electrophysiology add to the diagnosis of CIDP with reports fulfilling at least 3 of the 4 pre-established points.

**Table 1:** Electrophysiological characteristics in pre-referral underdiagnoses of CIDP

Pre-referral diagnosis	No. of pre-established electrophysiological points* present in pre-referral electrophysiology reports	Final diagnosis of CIDP in inflammatory neuropathy clinic
CIAP	1	Definite
CIAP	2	Definite
CIAP	1	Probable
CIAP	4	Definite
CNS inflammatory disease	2	Definite
CNS inflammatory disease	3	Definite
Diabetic neuropathy	1	Definite
Diabetic neuropathy	4	Definite
Diabetic neuropathy	1	Definite
Diabetic neuropathy	1	Probable
Diabetic neuropathy	1	Definite
Entrapment neuropathy	1	Definite
Entrapment neuropathy	1	Definite
Entrapment neuropathy	1	Definite
Genetic neuropathy	1	Definite
Genetic neuropathy	1	Definite
Genetic neuropathy	1	Definite
Genetic neuropathy	1	Probable
Genetic neuropathy	1	Definite
Genetic neuropathy	2	Definite
Genetic neuropathy	1	Definite
Genetic neuropathy	1	Definite
Lyme’s disease	1	Definite
Paraprotein-associated neuropathy	1	Probable
Stroke	2	Definite
Toxic neuropathy	4	Definite
Vasculitic neuropathy	1	Definite

*CIDP* chronic inflammatory demyelinating polyneuropathy, *CIAP* chronic idiopathic axonal polyneuropathy

*4 points based on EFNS/PNS criteria: (i) consideration of motor conduction slowing and distal motor latency prolongation for two nerves (ii) consideration of conduction block, temporal dispersion, F-wave analysis, for at least two nerves (iii) mention of using a set of electrodiagnostic criteria, or cut-offs to define significant abnormality (iv) mention of “acquired demyelinating neuropathy” or “CIDP

The mean delay in final diagnosis of CIDP, in the 27 patients with a pre-referral non-GBS underdiagnoses, was of 21.3 months (range 2–132 months); and mean delay to initiation of first attempted treatment for CIDP was 22.4 months (range 0–134 months). In comparison, for 19 patients with a pre-referral diagnosis of CIDP, there was no delay in diagnosis and initiation of first attempted treatment.

## Discussion

We investigated the frequency and causes of pre-referral underdiagnoses of CIDP in consecutive patients attending our specialist inflammatory neuropathy service, and also determined the diagnostic and treatment delay. This aspect of underdiagnoses in treatment responsive CIDP has been explored rarely [3, 5]. To the best of our knowledge, this is the first study focussing on specific clinical and electrophysiological features of CIDP contributing to the underdiagnoses, if unnoticed at the time of initial presentation.

In our cohort, 23.3% cases had a pre-referral diagnosis of GBS which was corrected to acute-onset CIDP. In comparison others have reported acute-onset CIDP cases to be around 16% [14]. As this presentation is unavoidable, therefore, we did not consider these patients as an underdiagnoses. We suggest that patients with acute onset polyradiculoneuropathy may require more active, vigilant and regular follow-up for timely identification of CIDP and intervention, through the rehabilitation phase after initial diagnosis.

Forty-five percent of patients had a pre-referral erroneous non-GBS underdiagnoses of non-inflammatory neuropathy, comparable to previous studies [8, 15, 16]. We believe that these underdiagnoses have multiple causes. For example, proximal weakness was the predominant feature in more than 70% of underdiagnosed patients, inadvertently missing this important clinical feature at the time of initial presentation could be an avoidable cause of underdiagnoses of treatment responsive CIDP. The lack of recognition of multifocal motor deficits and loss of proprioception in remaining 25% of patients in this cohort, in complex typical and atypical CIDP variants, can be another potential cause for CIDP underdiagnoses. Also, the frequency of final diagnosis of atypical forms of CIDP was higher in patients with pre-referral erroneous non-GBS underdiagnosis, as compared to patients with pre-referral CIDP diagnosis, although this was statistically non-significantly. It is possible that lack of awareness about atypical forms is adding to these CIDP underdiagnoses. In addition, a referral bias, from community to specialist centres, due to lack awareness and familiarity with complex atypical forms of CIDP e.g., distal and sensory CIDP variants may further contribute to CIDP underdiagnoses.

Our findings of CIDP co-existent with genetic neuropathy is in line with previous studies [17–19]. These findings highlight that unusual new clinical features which are unexpected in genetic neuropathies, such as rapid development of sensorimotor loss, proximal weakness and loss of proprioception, together with electrodiagnostic pointers of acquired inflammatory neuropathy, should raise the suspicion of a treatable inflammatory neuropathy.

In 85% of pre-referral non-GBS underdiagnoses, electrophysiology technical reports and their interpretations were contributing to the underdiagnoses. This may be, in part, due to non-inclusion of specific EFNS/PNS criteria derived parameters [6] as in this study. This can also potentially influence the requesting physician/clinical neurologist for eventual decision-making, leading to underdiagnoses as much as over diagnoses. These findings are in keeping with previous reports [20] on the undesirable consequences of limited electrophysiology interpretations in suspected CIDP cases.

Another reason for CIDP underdiagnoses can be potential lack of coordination between Neurologists and Neurophysiologists prior to the referral to the specialist centres. Albeit, in particular to the United Kingdom, electrophysiology is performed and reported in the overwhelming majority of cases by clinical neurophysiologists following referral from general physicians, Neurologists and other specialists. Hence, there could be a difference of opinion in interpretation of clinical and electrophysiological findings between different specialists. In this setting, the electrophysiology reports become essential for decision-making.

Our findings also point to the suggestion of lack of interest in existing EFNS/PNS Guidelines for inflammatory neuropathy, as demonstrated by a European Study Group on Guidelines for Neuropathy [21]. This highlights the need for simpler version of guidelines, their wider dissemination amongst training and general neurologists, and development of formal training programmes and courses in future.

CIDP underdiagnoses, may lead to quite wide-ranging delay in appropriate diagnosis and offering immunotherapeutic treatment as shown in our study, in line with previous studies [3, 5]. Moreover, axonal loss in CIDP worsens with time and adversely affects treatment response [22], again prompting the need for early diagnosis and intervention to limit the disability.

Our study has some limitations, including its single centre and retrospective design as well as limited number of patients included. We did not consider the diagnostic consequences of CSF protein levels, of MR imaging or somatosensory evoked potentials (SSEPs) in this study as we aimed primarily to look at the clinical and electrophysiological features which contributed to the underdiagnoses of CIDP prior to the referral to a specialist neuropathy clinic. Future studies, focussing on these additional parameters can further delineate the understanding of underdiagnoses in patients with CIDP. However, we believe that despite above-mentioned limitations, this study offers a perspective on the important clinical and electrophysiological features leading to CIDP underdiagnoses which can be addressed.

In conclusion, CIDP remains a rare disorder causing significant disability and impairment of quality of life. It is, however, most importantly treatable, which makes underdiagnoses and diagnostic delay highly undesirable. In view of our results, a focus of future disease guidelines and training on the frequently overlooked important clinical and electrophysiological aspects of the disease, appears essential for optimal care for patients with CIDP.
